# Usefulness of the Support Video “Talking Picture Book” for Overcoming Hesitancy to Start Galcanezumab Therapy

**DOI:** 10.1002/brb3.70447

**Published:** 2025-05-11

**Authors:** Hisanao Akiyama, Yoshihisa Yamano

**Affiliations:** ^1^ Department of Neurology St. Marianna University School of Medicine Kawasaki Kanagawa Japan

**Keywords:** anti‐CGRP antibody, galcanezumab, supportive video material, talking picture book

## Abstract

**Background:**

Galcanezumab, approved in April 2021, is the first humanized monoclonal antibody against the calcitonin gene‐related peptide (CGRP) ligand to become available in Japan, but some patients with migraine are hesitant to start an anti‐CGRP antibody for a variety of reasons, particularly its high price. To address this, a video in Japanese called “Talking Picture Book,” which aims to alleviate patients’ concerns about the anti‐CGRP antibody, was released as support material in Japan in March 2022.

**Method:**

Between December 2022 and May 2023, we conducted a questionnaire survey of 34 consecutive patients who had just viewed the video in order to evaluate its effectiveness in persuading hesitant patients to start an anti‐CGRP antibody.

**Result:**

Of these 34 patients, 79.8% gained a better understanding of migraine, 61.8% expressed a willingness to try galcanezumab, and 52.4% (11 patients) actually started the anti‐CGRP antibody. The number of mean monthly migraine days was significantly greater in those 11 patients than in the others. Multivariable logistic analysis of factors contributing to the decision to start anti‐CGRP antibody treatment revealed significant associations with monthly migraine days (odds ratio, 1.30; *p* = 0.01) and patients’ willingness to try anti‐CGRP antibodies (odds ratio, 9.82; *p* = 0.04).

**Conclusion:**

This material was particularly useful for patients with a high number of monthly migraine days who had been hesitant to start the therapy. “Talking Picture Book” is a useful material that complements physicians’ explanations of the therapy, and it is desirable that such video materials be adapted for use in other countries in the future.

## Graphical abstract

“Talking Picture Book” is a useful material that complements physicians. explanation of the therapy, and it is desirable that such video materials be adapted for use in other countries in the future.

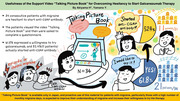

## 1 Introduction

Galcanezumab (Emgality) became the first humanized monoclonal antibody against the calcitonin gene‐related peptide (CGRP) ligand for migraine prevention therapy released in Japan when it launched in April 2021 and was followed by erenumab (Aimovig) and fremanezumab (Ajovy). The efficacy, speed of onset of efficacy, and safety of these three drugs, which are the features of migraine prevention therapy desired most by patients (Peres et al. 2007), have been confirmed by multiple large‐scale clinical studies (Skljarevski et al. 2018, Stauffer et al. 2018, Skljarevski et al. 2018, Detke et al. 2018, Camporeale et al. 2018, Sakai et al. 2020, Hirata et al. 2021, Mulleners et al. 2020, Goadsby et al. 2017, Sun et al. 2016, Tepper et al. 2017, Takeshima et al. 2021, Sakai et al. 2019, Silberstein et al. 2017, Dodick et al. 2018, Sakai et al. 2021), resulting in a paradigm shift in migraine prevention in the clinical setting. However, even when their efficacy, speed of onset of efficacy, and safety are explained by a physician, many patients with migraine are initially hesitant to start these drugs because of their high price, the route of administration (i.e., subcutaneous injection), and doubts about the efficacy, speed of onset of efficacy, and safety, based on their previous experience with conventional preventive treatments.

**FIGURE 1:**
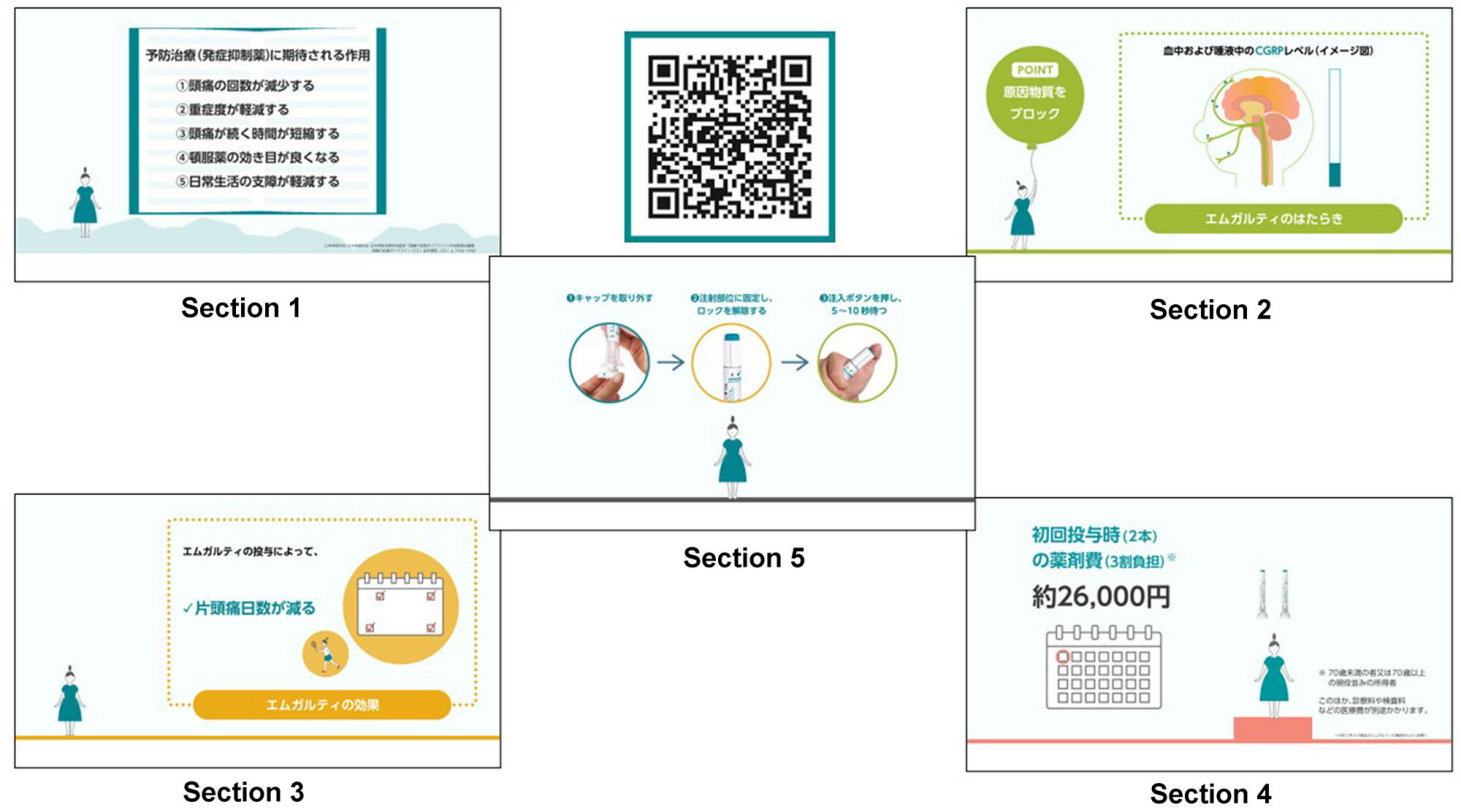
Five sections of “Talking Picture Book”. “Talking Picture Book” comprises five sections: (1) What can be expected from preventive treatments; (2) How it works and how it will be administered; (3) Effects and possible adverse effects; (4) Evaluation of efficacy; and (5) Two methods of administration.

To alleviate patients’ concerns about starting an anti‐CGRP antibody, physicians need to spend adequate time to explain the benefits of the therapy, which include improving various aspects of daily life as well as financial hardship due to missed work, but this is not always possible in the clinical setting. To address this situation, “Talking Picture Book,” supportive video material in Japanese aimed at alleviating patients’ concerns about starting the therapy, was developed by the supplier of galcanezumab and was released in March 2022 (Figure 1).

We recruited patients who were hesitant about starting anti‐CGRP antibody after receiving therapy information from a physician and conducted a questionnaire survey immediately after they viewed the video “Talking Picture Book,” which is available only in Japan, in order to investigate the effectiveness of this material. The purpose of this study is to describe patients’ rating of usefulness for the video material “Talking Picture Book” in different domains and also demographic factors that predict willingness to consider or to actually commence anti‐CGRP antibody.

## 2 Participants and Methods

### 2.1 Participants

Participants were 34 consecutive patients with migraine for whom use of the anti‐CGRP antibody (galcanezumab or fremanezumab) and the fully human monoclonal antibody CGRP receptor antagonist (erenumab) was indicated by the “Optimal Use Promotion Guidelines,” but who were hesitant to start the therapy for a variety of reasons. First, the participants viewed the video material “Talking Picture Book” and then were asked to complete a questionnaire survey about the contents of the video between December 2022 and May 2023. The participants’ characteristics (age, sex, years of migraine history, monthly migraine days) contained in their medical records were examined retrospectively.

The survey comprised the following questions: (1) “Did your understanding of migraine improve after viewing the video?” (“Yes” or “No”); (2) “Would you like to try galcanezumab (Emgality) after viewing the video?” (“Yes” or “No”); (3) “Did you learn something new from the video?” (“Yes” or “No”); (4) Which section(s) of the video left the strongest impression? (Section(s) 1, 2, 3, and/or 4; choose all that apply [Note: Section 5 was added later and was therefore not evaluated in this study]).

### 2.2 “Talking Picture Book”

The “Talking Picture Book,” developed by Eli Lilly Japan K.K. and Daiichi Sankyo Co., Ltd. in Japan, is a short video that explains galcanezumab in plain language to patients with migraine to help them better understand the benefits of the anti‐CGRP antibody. It is expected to assist physicians in obtaining informed consent from patients who are hesitant to start the therapy. When it first became available in March 2022, it consisted of the following four sections: (1) What can be expected from preventive treatments (2 min 34 s); (2) How it works and how it will be administered (1 min 45 s); (3) Effects and possible adverse effects (1 min 32 s); and (4) Evaluation of efficacy (2 min 3 s). In May 2022, a fifth section (5) Two methods of administration (2 min 37 s) were added, resulting in a total video length of 10 min 31 s (Figure 1). “Talking Picture Book” was designed to be viewed by patients together with a physician and/or a nurse in a consultation room, waiting room, or treatment room at a medical facility after receiving general information about migraine treatment from a physician. However, today, patients can view the video anywhere at their leisure by scanning a QR code (Figure 1) with their smartphone.

### 2.3 Ethical Considerations and Consent to Participate

The study was performed according to the guidelines of the Helsinki Declaration and was approved by the Institutional Medical Ethics Committee of the St. Marianna University School of Medicine Bioethics Committee (approval no. 5891). All patients provided written informed consent to participate in this study. Permission to use the copyright of the “Talking Picture Book” has been obtained from Eli Lilly Japan K.K.

### 2.4 Statistical Analysis

Data are presented as means and standard deviations. Unpaired Student's *t*‐test was used to compare age, duration of migraine, and number of migraine days per month, while the chi‐square test was used to compare differences according to sex in the responses to each question between the two groups. Because this was confirmatory research with a small number of cases and no previous research, we created three models and performed multivariable logistic analysis. The multivariable logistic analysis was used to assess associations of introducing anti‐CGRP antibodies with age, duration of migraine headache, number of migraine days per month, understanding of migraine, and willingness or desire to try anti‐CGRP antibodies. Odds ratios and 95% confidence intervals (CI) were calculated for each model group. All statistical analyses were conducted using IBM SPSS Statistics for Windows ver. 26 (Chicago, IL). All statistical tests were two‐sided, and *p*‐values <0.05 were considered statistically significant.

## 3 Results

### 3.1 Patient Characteristics

Of the 34 patients who viewed the video material “Talking Picture Book,” 30 were women. The mean age of the participants was 45.4 ± 11.3 years (range, 24–74 years), the mean years of migraine history was 24.3 ± 12.2 years (range, 6–49 years), and the mean number of monthly migraine days was 9.9 ± 6.2 days (range, 3–25 days).

### 3.2 Postviewing Questionnaire Survey

#### 3.2.1 Improved Understanding of Migraine

To the question “Did your understanding of migraine improve after viewing the video?” 27 patients (79.4%) answered “Yes,” and 7 (20.6%) answered “No”. The number of women (24 vs. 6), mean age (44.9 ± 10.3 years vs. 47.6 ± 14.4 years), mean years of migraine history (24.1 ± 12.1 years vs. 25.3 ± 12.5 years), and mean number of monthly migraine days (10.6 ± 6.7 days vs. 7.3 ± 2.8 days, *p* = 0.07) are not significant in those who answered “Yes” compared with those who answered “No”.

#### 3.2.2 Willingness to Start Galcanezumab (Emgality)

**FIGURE 2:**
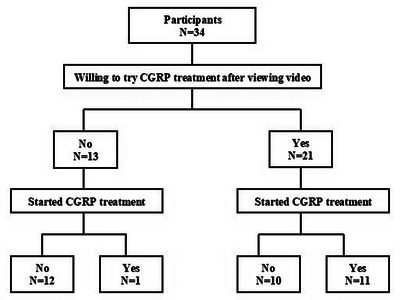
Flow chart for the participants to start anti‐CGRP antibody. To the question “Would you like to try galcanezumab (Emgality) after viewing the video?” 21 patients answered “Yes” and 13 answered “No.” 11 of the 21 patients who answered “Yes” actually commenced treatment (9 galcanezumab; 2 fremanezumab) and 1 of the 13 patients who answered “No” (*p* = 0.01, Fisher's exact test).

**FIGURE 3:**
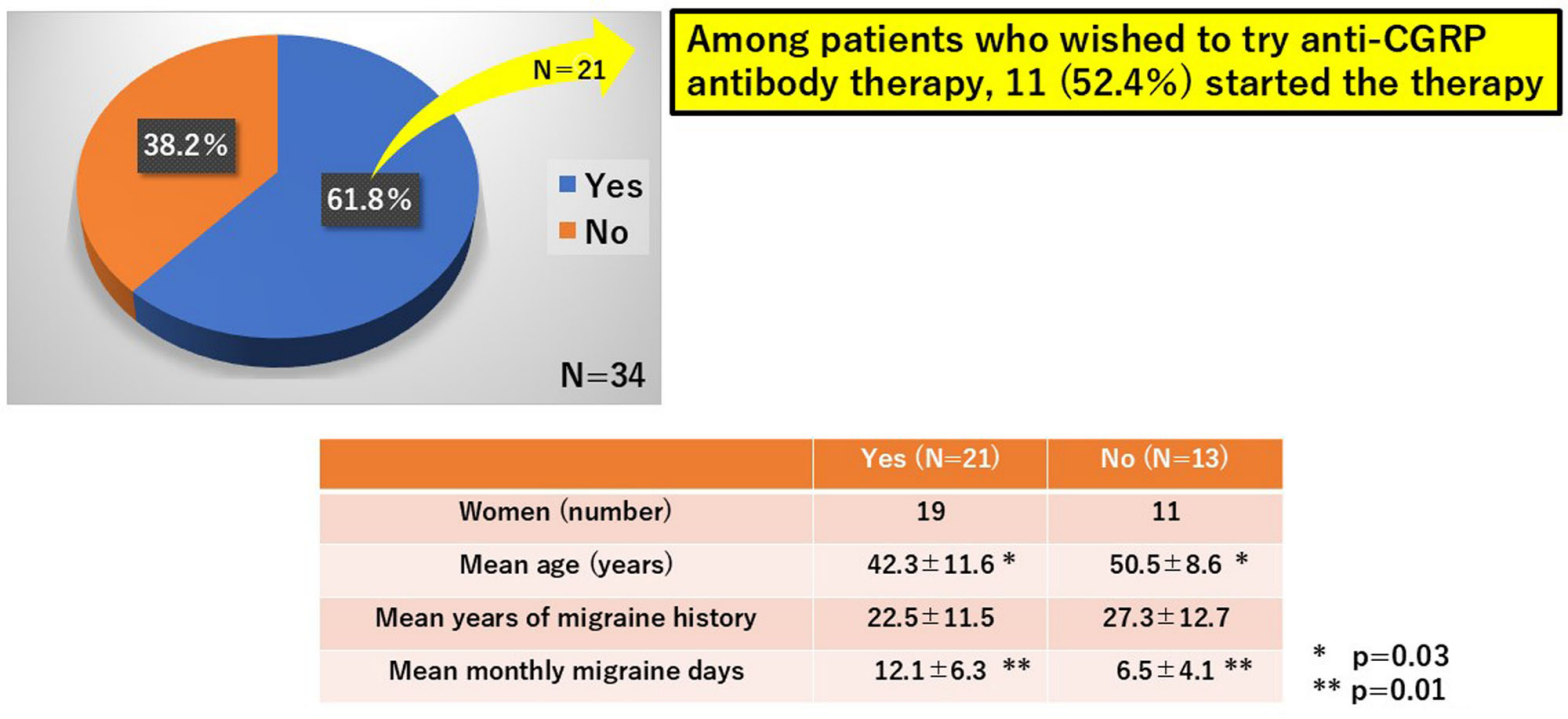
Willingness to start galcanezumab (Emgality) after viewing “Talking Picture Book”. To the question “Would you like to try galcanezumab (Emgality) after viewing the video?” 21 patients (61.8%) answered “Yes” and 13 (38.2%) answered “No.” There were significant differences in mean age (*p* = 0.03) and mean monthly migraine days (*p* = 0.01) between those who answered “Yes” and those who answered “No.”

To the question “Would you like to try galcanezumab (Emgality) after viewing the video?” 21 patients (61.8%) answered “Yes” and 13 (38.2%) answered “No” (Figures 2, 3). There were significant differences in mean age (42.3 ± 11.6 years vs. 50.5 ± 8.6 years, *p* = 0.03) and mean number of monthly migraine days (12.1 ± 6.3 days vs. 6.5 ± 4.1 days, *p* = 0.01) between those who answered “Yes” and those who answered “No.” In other words, migraine patients who answered they would like to try galcanezumab (Emgality) tended to be younger and have more migraine days. There was also a significant difference in the proportion of patients who started anti‐CGRP antibody after viewing the video: 11 of the 21 patients (52.4%) who answered “Yes” actually commenced treatment (9 galcanezumab; 2 fremanezumab) compared with 1 of the 13 patients (7.7％) who answered “No” (*p* = 0.01, Fisher's exact test) (Figure 2).

When comparing the 11 patients who started anti‐CGRP antibody and the 10 who did not among the 21 patients who answered “Yes,” the mean number of monthly migraine days was significantly greater in the former than in the latter (14.9 ± 5.2 days vs. 9.0 ± 6.0 days, *p* = 0.04), but there were no significant differences in sex, mean age, and mean years of migraine history. Multivariable logistic analysis revealed that monthly migraine days (odds ratio, 1.30; *p* = 0.01) and patients’ willingness to try anti‐CGRP antibody (odds ratio, 9.82; *p* = 0.04) were significant factors associated with starting anti‐CGRP antibody (Table 1).

**TABLE 1:** Multivariable logistic analysis for patients who started anti‐CGRP antibody.

	Odds ratio	95% CI	*p*‐value
Multivariable analysis (Model 1)			
Age	0.93	0.84−1.04	0.21
Years of migraine history	1.06	0.96−1.17	0.29
Monthly migraine days	1.30	1.08−1.57	0.01
Multivariable analysis (Model 2)			
Age	0.92	0.83−1.01	0.06
Years of migraine history	1.06	0.95−1.12	0.50
Understanding of migraine	1.47	0.19−9.87	0.76
Multivariable analysis (Model 3)			
Age	0.94	0.86−1.04	0.21
Years of migraine history	1.03	0.94−1.13	0.55
Willingness to try an anti‐CGRP antibody	9.82	1.00−96.05	0.04

*Note*: The multivariable logistic analysis was used to assess associations of introducing anti‐CGRP antibodies with age, duration of migraine headache, number of migraine days per month, understanding of migraine, and willingness or desire to try anti‐CGRP antibodies. (Model 1: age, years of migraine history, and monthly migraine days; Model 2: age, years of migraine history, and understanding of migraine; Model 3: age, years of migraine history, and willingness to try an anti‐CGRP antibody). Multivariable logistic analysis revealed that monthly migraine days (odds ratio, 1.30; *p* = 0.01) and patients’ willingness to try anti‐CGRP antibody (odds ratio, 9.82; *p* = 0.04) were significant factors associated with starting anti‐CGRP antibody.

Abbreviations: 95% CI; 95%confidence interval.

#### 3.2.3 New Information

To the question “Did you learn something new from the video?” 14 patients (41.2%) answered “Yes” and 20 (58.8%) answered “No.” There were no significant differences in sex women (19 vs 11), mean age (42.3 ± 11.6 years vs. 50.5 ± 8.6 years), mean years of migraine history (22.5 ± 11.5 years vs. 27.3 ± 12.7 years), and mean monthly migraine days (12.1 ± 6.3 days vs. 6.5 ± 4.1 days) between the two groups. Information such as free comments that were new to the patients included the mechanism of migraine onset, the mechanism of action of anti‐CGRP antibodies, the reason for the high price of galcanezumab, and the efficacy and safety of galcanezumab.

#### 3.2.4 Section of the Video That Left the Strongest Impression

To the question “Which section of the video‐information such as how to administer the drug and information on potential side effects, left the strongest impression (multiple choice allowed)?” 11 patients (32.4%) chose Section 1 (What can be expected from preventive treatments), 21 (61.8%) chose Section 2 (How it works and how it will be administered), 19 (55.9%) chose Section 3 (Effects and possible adverse effects), and 41.2% chose Section 4 (Evaluation of efficacy), indicating that Sections 2 and 3 left a strong impression.

## 4 Discussion

Migraine is a neurological disorder that has a considerable impact on activities of daily living and can, therefore, affect a person's ability to work. Migraine treatment involves two components: acute and preventive therapy. However, conventional preventive drugs were not developed as targeted migraine treatments and often exert nonspecific effects on a range of molecular targets; thus, their efficacy, speed of onset of efficacy, and safety are not satisfactory.

In Japan, three anti‐CGRP or CGRP receptor antibody drugs were successively launched starting in April 2021. The efficacy, speed of onset of efficacy, and safety of these drugs have been demonstrated in the clinical setting and confirmed by large‐scale clinical studies: galcanezumab by the CGAB, CGAG, CGAH, CGAI, and CGAJ studies conducted outside Japan, the CGAN and CGAP studies conducted domestically, and the CGAW study conducted through domestic–international collaboration; erenumab by the STRIVE study conducted outside Japan; and fremanezumab by the HALO EM and HALO CM studies conducted outside Japan, and a study involving Japanese and Korean patients (Skljarevski et al. 2018, Stauffer et al. 2018, Skljarevski et al. 2018, Detke et al. 2018, Camporeale et al. 2018, Sakai et al. 2020, Hirata et al. 2021, Mulleners et al. 2020, Goadsby et al. 2017, Sun et al. 2016, Tepper et al. 2017, Takeshima et al. 2021, Sakai et al. 2019, Silberstein et al. 2017, Dodick et al. 2018, Sakai et al. 2021).

One of the challenges in the clinical setting is that physicians have limited consultation time and might not be able to provide an adequate explanation, and patients remain hesitant to start the anti‐CGRP antibody because they are skeptical of its efficacy, speed of onset of efficacy, and safety because of their previous experience with conventional preventive medicines (Peres et al. 2007). Furthermore, patients are concerned about the high price—especially for galcanezumab, which requires a loading dose of 240 mg, and costs approximately 26,000 yen (USD 173.33, at an exchange rate of JPY 150 to USD 1)—and the administration by subcutaneous injection, which is the first of its kind for migraine prevention. “Talking Picture Book” was developed to address these concerns to the extent possible as well as to facilitate the informed consent process. At present, this video material is available only in Japan.

In this study, participants were patients who were recommended anti‐CGRP antibody (galcanezumab in most cases) by their physician. A questionnaire survey was administered just after the patient viewed the video “Talking Picture Book.” It was revealed that about 80% of the patients, especially those with a relatively high number of monthly migraine days, improved their understanding of migraine, indicating the effectiveness of this material. We observed that the majority of people (21 vs. 13) expressed a willingness to try galcanezumab after viewing the video. Those who expressed a willingness tended to be younger and experienced a higher number of monthly migraine days. However, of those that initially appeared willing, only about half actually commenced treatment, with no significant difference in age observed between those commencing treatment and those that did not. The number of monthly migraine days was also significantly greater in patients who actually started any anti‐CGRP antibody compared with those who did not. Overall, our findings suggest that patients with a high number of monthly migraine days, including younger people, appear more receptive to starting anti‐CGRP antibody; however, there may also be barriers to treatment, including affordability and the difficulties in making routine hospital visits due to work.

This study also demonstrated the types of information that were not well understood by patients prior to viewing the video material. Among patients who improved their understanding of migraine (ranging from the mechanism of migraine to their treatment) and expressed a willingness to start galcanezumab, 52.4% actually started the therapy. It is difficult to judge whether 52.4% is high or low, but this study showed that mean monthly migraine days and patients’ willingness to try anti‐CGRP antibodies significantly contributed to the initiation of therapy. Although the rate of anti‐CGRP antibody induction even without viewing the video material is unknown and cannot be compared, it is likely that viewing this video material is critical for overcoming hesitancy to try anti‐CGRP antibodies in patients with a high number of monthly migraine days. Also, some patients were likely to remain hesitant if they did not view the video, suggesting that this material would contribute to increasing the number of patients with migraine who would benefit from anti‐CGRP antibodies.

Patients were most interested in the “How it works and how it will be administered” and “Effects and possible adverse effects” sections of the video, indicating their interest in the efficacy, speed of onset of efficacy, and safety of galcanezumab. Many physicians pointed out the high prices of anti‐CGRP antibodies as a reason for patients’ hesitancy to start the therapy. The “Talking Picture Book” video clearly explains that anti‐CGRP antibodies are expensive because they are biologically synthesized and, unlike chemically synthesized products, cannot be mass‐produced in pharmaceutical factories. It is likely that patients who understood and accepted this explanation were more willing to start the therapy.

This study has several limitations. First, it was a single‐center questionnaire survey with a small sample size, and the majority of participants were women, reflecting the nature of migraine. A multicenter survey involving a larger number of participants with a more balanced sex ratio should be conducted in the future. Second, we do not have any data on the number of patients routinely offered anti‐CGRP antibody in a clinic, what proportion actually start anti‐CGRP antibody. In the same vein, while these are hesitant patients, it is not clear whether a similar number would have opted for treatment if given sufficient time to consider it—therefore, it is difficult to know the added value of the intervention (video) on the initiation of anti‐CGRP antibodies. Third, we did not explore free text comments, which may give additional information that initially expressed a willingness to not take up the offer of treatment.

## 5 Conclusion

Anti‐CGRP antibodies are now essential for the prevention of migraine. However, at present, not every patient with migraine is benefitting from these drugs, indicating the need for solutions that alleviate the concerns of patients who remain hesitant to start the therapy even after receiving information from a physician. At present, “Talking Picture Book” is available only in Japan, and proactive use of this material for patients with migraine, particularly those with a high number of monthly migraine days, is expected to improve their understanding of migraine and increase their willingness to try the therapy. It is hoped that at least half of these patients will start anti‐CGRP antibody irrespective of the particular drug, and thus it is desirable to adapt the “Talking Picture Book” for use in other countries in the future.

## Author Contributions

Hisanao Akiyama examined the patient and drafted the manuscript. Yoshihisa Yamano helped to draft the manuscript. All authors read and approved the final manuscript.

## Ethics Statement

The study was approved by the Institutional Medical Ethics Committee of the St. Marianna University School of Medicine Bioethics Committee (approval no. 5891).

## Informed Consent

All patients provided written informed consent to participate in this study.

## Conflicts of Interest

Author H.A. received speaker honoraria from Daiichi Sankyo Co., Ltd, Eli Lilly Japan K.K., Otsuka Pharmaceutical Co., Ltd, and Amgen Inc, but did not receive payment for writing manuscript from them.

### Peer Review

The peer review history for this article is available at https://publons.com/publon/10.1002/brb3.70447

## Data Availability

The datasets used and analyzed during this study are available from the corresponding author upon reasonable request.
